# Intrinsic Tau Acetylation Is Coupled to Auto-Proteolytic Tau Fragmentation

**DOI:** 10.1371/journal.pone.0158470

**Published:** 2016-07-06

**Authors:** Todd J. Cohen, Brian H. Constance, Andrew W. Hwang, Michael James, Chao-Xing Yuan

**Affiliations:** 1 Department of Neurology, UNC Neuroscience Center, University of North Carolina, Chapel Hill, North Carolina, United States of America; 2 Department of Pharmacology, University of North Carolina, Chapel Hill, North Carolina, United States of America; 3 Department of Pathology and Laboratory Medicine, Institute on Aging and Center for Neurodegenerative Disease Research, University of Pennsylvania School of Medicine, Philadelphia, Pennsylvania, United States of America; 4 Department of Pharmacology, University of Pennsylvania School of Medicine, Philadelphia, Pennsylvania, United States of America; New York State Institute for Basic Research, UNITED STATES

## Abstract

Tau proteins are abnormally aggregated in a range of neurodegenerative tauopathies including Alzheimer’s disease (AD). Recently, tau has emerged as an extensively post-translationally modified protein, among which lysine acetylation is critical for normal tau function and its pathological aggregation. Here, we demonstrate that tau isoforms have different propensities to undergo lysine acetylation, with auto-acetylation occurring more prominently within the lysine-rich microtubule-binding repeats. Unexpectedly, we identified a unique intrinsic property of tau in which auto-acetylation induces proteolytic tau cleavage, thereby generating distinct N- and C-terminal tau fragments. Supporting a catalytic reaction-based mechanism, mapping and mutagenesis studies showed that tau cysteines, which are required for acetyl group transfer, are also essential for auto-proteolytic tau processing. Further mass spectrometry analysis identified the C-terminal 2^nd^ and 4^th^ microtubule binding repeats as potential sites of auto-cleavage. The identification of acetylation-mediated auto-proteolysis provides a new biochemical mechanism for tau self-regulation and warrants further investigation into whether auto-catalytic functions of tau are implicated in AD and other tauopathies.

## Introduction

Tau proteins are expressed primarily in the nervous system and are comprised of six isoforms containing up to two N-terminal repeats (0N, 1N, or 2N) and either three (3R-tau) or four (4R-tau) repeat domains that contribute to tau-microtubule (MT) binding, thereby regulating MT stability [1, 2]. We and others previously demonstrated that tau is extensively acetylated on lysine residues mainly residing within the MT-binding repeats (MTBR), thus providing a novel regulatory modification controlling normal and abnormal tau properties [3–5]. Functional studies showed that tau acetylation impaired normal tau-MT interactions, prevented physiological tau-mediated stabilization of MTs, and altered pathological tau fibril formation that is predominantly associated with insoluble, Thioflavin-positive tau aggregates [3, 5]. Indeed, the disease relevance of tau acetylation was demonstrated in neuropathological and biochemical analysis of a panel of human tauopathy cases. Acetylation at residue K280 (Lys280) showed a distinctly pathological signature marking mature tau lesions in Alzheimer’s disease (AD), corticobasal degeneration (CBD), progressive supranuclear palsy (PSP), and several FTDP-17 familial cases of dementia [3] but was rarely observed in control brain tissue or cultured wild-type cells or neurons [4], illustrating the disease-specific nature of K280 acetylation.

More recently, tau acetylation at other critical residues including K174, K274, and K281 has been shown to promote AD-related cognitive deficits, synaptic defects, and impaired hippocampal long-term potentiation (LTP) [6, 7], strongly implicating tau acetylation in AD pathogenesis. While the specific pathogenic signaling pathways mediated by acetylated tau are emerging [7], the relationship of tau acetylation to other disease-associated tau modifications (e.g. phosphorylation, ubiquitination, and proteolytic cleavage) is not well understood. However, previous *in vitro* studies as well as proteomic analysis in mouse brain suggests a global tau acetylation profile that overlaps with known sites of tau ubiquitination [5, 8], implying PTM competition could dictate tau function. Ongoing efforts to dissect tau post-translational processing could provide a step-wise framework for tau pathogenesis.

While previous studies have suggested tau acetylation occurs *in trans* by Creb-binding protein (CBP/p300) and possibly other yet-to-be-identified acetyltransferases [3, 5, 9, 10], evidence also indicates that tau auto-acetylation can occur upon incubation of tau proteins with acetyl-CoA alone. Indeed, many acetyltransferases control their own catalytic activity via positive feedback auto-acetylation [11–16]. We proposed that tau utilizes a cysteine-mediated acetyl group transfer onto its lysine residues [9], which is consistent with the mechanism proposed for MYST and N-arylamine (NAT) acetyltransferases [17, 18], to which tau has some functional and sequence similarities [9]. This acetyl transfer mechanism from cysteine to lysine residues contrasts with previously reported non-specific acetylation of cysteines observed with *in vitro* peptide substrates, which can often lead to false positive assignments of lysine acetylation [19]. Supporting cysteine-mediated tau auto-acetylation, a recent molecular simulation study of tau suggested close cysteine-lysine distances that could facilitate self-acetylation [20]. Remarkably, a recent profiling study indicated that auto-acetylation of cellular proteins could even occur in the apparent absence of enzymatic activity [21], in which case lysine specificity may be dictated by lysine accessibility and/or specific lysine pKa values. Such non-enzymatic auto-acetylation is a prominent feature of mitochondria localized proteins, where acetyl-CoA levels are highly enriched [22–24].

We investigated the impact of tau acetylation using recombinant tau proteins with a variable number of N-terminal inserts or C-terminal MTBR domains. Surprisingly, we found that auto-acetylation occurs within the MTBR region, while CBP-mediated acetylation occurs both within the MTBR and also distally in the proline-rich region. To assess the regulatory function of tau auto-acetylation, *in vitro* assays revealed an unexpected auto-proteolytic cleavage event dependent on tau catalytic cysteine residues (C291/C322) residing within the MTBR, leading to the production of distinct N- and C-terminal tau fragments. Our study suggests that tau auto-acetylation may be coupled to downstream self-degradation mechanisms, which could be relevant to AD and related tauopathies characterized by tau hyper-acetylation and pathological cleavage fragments.

## Materials and Methods

### Plasmids and Cell Culture

All human tau isoforms containing 0, 1, or 2 N-terminal inserts and either 3 or 4 MTBR regions were cloned into pCDNA5/TO vector (Invitrogen) and site-directed mutagenesis (Quikchange kit; Agilent, Santa Clara, CA) was used to create cysteine mutations at residues C291 and C322, where indicated. A mammalian expression plasmid containing wild-type CBP or the inactive L1435A/D1436A mutant (pcDNA3.1-CBP and CBP-LD) were kindly provided by Dr. Tso-Pang Yao (Duke University). All described plasmids were transfected into QBI-293 cells using Fugene (Roche) or Lipofectamine 2000 (Life Technologies) per the manufacturers detailed protocols.

### Biochemical extraction of cultured cells

Fractionation of all cell lysates was performed by sequential extraction using buffers of increasing strength. Cells from 6-well culture dishes were scraped into 300 μl RIPA buffer (50 mM Tris pH 8.0, 150 mM NaCl, 1% NP-40, 5 mM EDTA, 0.5% sodium deoxycholate, 0.1% SDS) containing 1 mM phenylmethylsulfonyl fluoride (PMSF), a mixture of protease inhibitors (1 μM pepstatin, leupeptin, *N*-p-Tosyl-l-phenylalanine chloromethyl ketone, Nα-Tosyl-l-lysine chloromethyl ketone hydrochloride, trypsin inhibitor; Sigma), and a mixture of phosphatase inhibitors (2 mM imidazole, 1 mM NaF, 1 mM sodium orthovanadate; Sigma). Samples were sonicated and centrifuged at 100,000xg for 30 min at 4°C, and then re-extracted in RIPA buffer to ensure complete removal of soluble proteins. Resultant insoluble pellets were extracted in 100 μl urea buffer (7 M urea, 2 M Thiourea, 4% CHAPS, 30 mM Tris, pH 8.5) containing the same formulation of inhibitors as described for RIPA buffer, then sonicated and centrifuged at 100,000xg for 30 min at room temperature. Soluble and insoluble fractions were analyzed by western blotting using the indicated antibodies. Tau antibodies used for western analysis were as follows: C-terminal T46 (1:1000, Thermofisher), tau-5 (1:1000, Thermofisher), tau-1 (1:1000, Thermofisher), K9JA (1:10,000, Dako), ac-K280 [3] (1:1000), and ac-K163 (1:1000, this study).

### Recombinant tau *in vitro* methods

#### Tau protein preparations

All full-length (0N4R, 1N4R, 2N4R, 0N3R, 1N3R, 2N3R), tau-K18 (4R), and tau-K19 (3R) proteins were purified using an AKTA-Pure FPLC chromatography system (GE). The proteins were expressed in Escherichia coli BL21(DE3) RIL strain. Bacteria were grown in LB media containing ampicillin at 37°C and induced with isopropyl-D -thiogalactopyranoside at a final concentration of 0.8 mM when the OD reached 0.6. After agitation for 2 h, cells were harvested by centrifugation. The pellet was re-suspended in high-salt RAB buffer [0.1 M MES, 1 mM EGTA, 0.5 mM MgSO_4_, 0.75 M NaCl, 0.02 M NaF, 0.1 mM PMSF, 0.1% protease inhibitor cocktail (100 μg/mL each of pepstatin A, leupeptin, TPCK, TLCK, and soybean trypsin inhibitor and 100 mM EDTA), pH 7.0] and homogenized. The cell lysates were heated to 100°C for 10 min, rapidly cooled on ice for 20 min and centrifuged at 70,000xg for 30 min. Supernatants were dialyzed into FPLC buffer A [20 mM piperazine-N, N-bis(2-ethanesulfonic acid) pH 6.5, 10 mM NaCl, 1 mM EGTA, 1mM MgSO_4_, 2 mM DTT, 0.1 mM PMSF], applied onto a HiTrap Sepharose HP IEX cation-exchange column (GE Healthcare), and eluted with a 0–0.4 M NaCl gradient using an AKTA-Pure FPLC system (GE Healthcare). The fractions were checked for the presence of the tau proteins by sodium dodecyl sulfate-polyacrylamide gel electrophoresis (SDS- PAGE) followed by Coomassie Blue R-250 staining. The fractions containing enriched purified tau were pooled together and dialyzed against 100 mM sodium acetate buffer, pH 7.0. Importantly, all purified tau proteins were subject to a critical heat-denaturation step for 30 min at 100° to inactivate any endogenous bacterial enzymes and simultaneously enrich for heat-stable tau proteins. Proteins were concentrated using Amicon Ultra centrifugal filter units (Millipore Corporation, Billerica, MA), and protein concentrations were determined using the bicinchoninic acid protein assay (Pierce, Rockford, IL) with bovine serum albumin as the protein standard.

#### *In vitro* acetylation reactions

Tau K18 or full-length tau proteins were either mock acetylated with coenzyme A alone or modified *in vitro* using acetyl-CoA, butyryl-CoA, or propionyl-CoA (Sigma) in acetylation reaction buffer (50 mM Tris–HCl pH 8.0, 10% glycerol, 1 mM DTT, 100 μM EDTA, 1 mM PMSF, 0.4 mM CoA derivative) at 37°C for the indicated timepoints from 1 day—7 days. Purified recombinant CBP protein (Enzo Life Sciences) (0.5 ug) was incubated in the acetylation reactions, where indicated, leading to maximal tau acetylation. Reaction products were analyzed by resolving proteins on 15% SDS-PAGE gels followed by Coomassie staining or subsequent immunoblotting with the indicated tau antibodies, where indicated. For tau pharmacological inhibition studies, tau proteins were pre-incubated on ice with 100 μM chymostatin, 1 mM N-ethylmaleimide (NEM), 4mM iodoacetamide (IA), 10 μM ALLN, and 10 μM Antipain (Sigma) followed by the above described acetylation reactions. All reactions were performed with a minimum of N = 3 independent experimental replicates. For tau fragmentation studies, error bars represent standard deviation (SD) among replicates and statistical calculations were performed using student t-test with significant p-values ≤ 0.01.

#### *In vitro* calpain reactions

Calpain protease digestions were performed using 20 μM recombinant tau (2N4R) and increasing concentrations of 0–0.5 Units recombinant calpain-2 (Millipore) and incubated at 30° for 10-min in calpain reaction buffer (50mM Tris, pH 7.5, 2mM DTT, 1mM EDTA, 100mM NaCl, 3mM CaCl_2_) followed by 5-min heat denaturing at 95° in SDS sample buffer and analysis of N- and C-terminal tau epitopes by immunoblotting.

#### Mass spectrometry

NanoLC nanospray MS-MS analysis was performed at the University of Pennsylvania proteomics core facility, which identified butyrylated and propionylated lysine residues. Total tau proteins (1μg total protein) and 0.4 mM butyryl or propionyl-CoA were incubated in 30 μl of reaction buffer (50 mM Tris-HCl pH 8.0, 10% glycerol, 1 mM DTT, 100 mM EDTA, 1 mM phenylmethylsulfonyl fluoride) for 1 hr at 37°C. Acylated tau products were analyzed by SDS-PAGE and Coomassie staining followed by gel excision for mass spectrometry using LTQ XL* Linear Ion Trap Mass Spectrometer (Thermo Scientific). Data was acquired using Xcalibur software (Thermo Scientific) and analyzed using Mascot, Scaffold, and PEAKS softwares.

### Size-exclusion Chromatography (SEC)

SEC was performed using an Acquity UPLC system equipped with a photodiode array detector (Waters Corp., Milford, MA). Injections of 15 μl were separated with an Acquity BEH200 SEC 1.7 μm (4.6 × 300 mm including a 4.6 × 30 guard column) using 100 mm sodium acetate, pH 5, with 300 mm NaCl at 0.3 ml/min over 30 min. Sample peaks were detected and analyzed using absorbance at 220 nm.

### Peptide/Antibody generation

**Fig 1:**
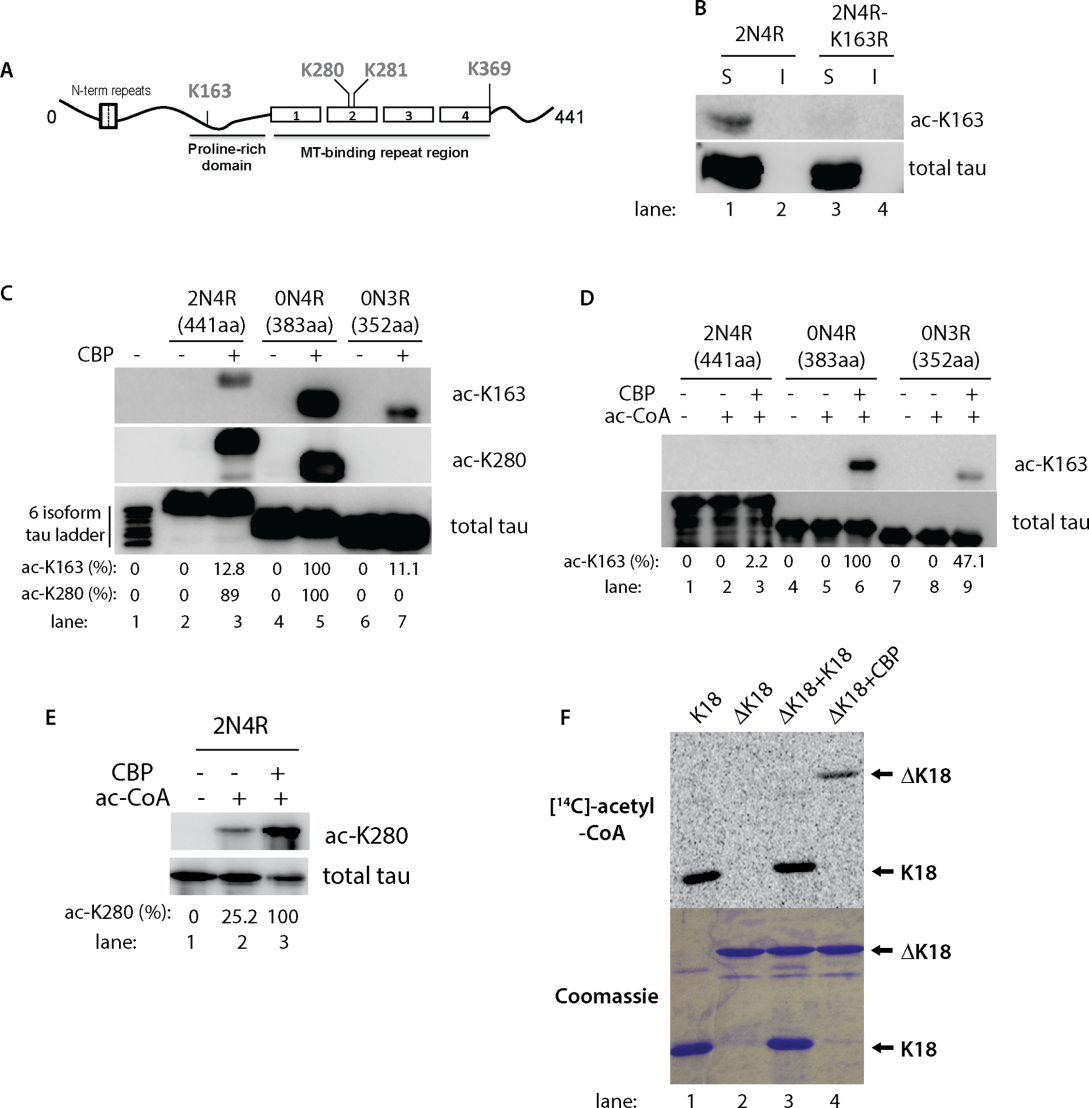
Tau isoforms display distinct patterns of acetylation at lysine residues K163 and K280. **A)** A schematic of the tau protein is shown depicting the microtubule-binding region (MTBR) and the proline-rich domain containing previously identified sites of tau acetylation. **B)** QBI-293 cells co-transfected with CBP and either WT or K163R mutant versions of 2N4R-tau were fractionated into RIPA soluble (S) and insoluble (I) fractions followed by immunoblotting with ac-K163 and K9JA antibodies. **C)** QBI-293 cells were transfected with 2N4R, 0N4R, or 0N3R expressing tau plasmids and cell lysates were immunoblotted using ac-163, ac-K280, and total tau K9JA antibodies. A six tau isoform ladder (lane 1) was included as a tau molecular weight marker and negative control, which showed no immunoreactivity with either acetylated tau antibody, as expected. **D)** Recombinant full-length tau isoforms (2N4R, 0N4R, 0N3R) were acetylated by incubation with acetyl-CoA in the presence or absence of CBP. Reaction products were subjected to immunoblot analysis using the acetylation-specific antibody against residue K163 (ac-K163) or K9JA to detect total tau proteins. **E)** Recombinant full-length tau 2N4R-tau was similarly incubated in acetylation reactions containing acetyl-CoA and/or CBP and reactions products were analyzed by immunoblotting with ac-K280 and K9JA antibodies. Ac-K163 and ac-K280 immunoreactivity was quantified, where indicated, using densitometry readings of acetylated tau protein bands. The percentage of acetylated tau was normalized to total tau and represented as a ratio of acetylated tau/total tau. **F)** *In vitro* acetylation reactions were performed similar to **(E)** above using tau-K18 (MTBR only) and tau-ΔK18 (full-length tau lacking the 4-repeats) incubated in the presence of purified CBP, where indicated, along with radiolabeled [^14^C]-acetyl-CoA. Acetylation reactions were analyzed by SDS-PAGE and Coomassie staining followed by autoradiography using STORM phosphorimager software to visualize acetylated tau protein bands.

Polyclonal anti-acetyl tau K280 antibodies were previously described [3]. Anti-acetyl tau K163 antibodies were generated using the acetylated tau peptide C-PGQKGQA containing K163 chemical acetylation to immunize rabbits (Pocono Rabbit Farm and Laboratory Inc., Canadensis, PA). Double affinity purification was performed using native and acetylated peptides sequentially using Sulfolink columns (Pierce Biotechnology). Site specificity of ac-K163 was confirmed using a K163R mutant tau that was unable to become fully acetylated in the presence of CBP (Fig 1).

### Brain tissue extraction and analysis

All brain tissue was acquired from the Center for Neurodegenerative Disease Research (CNDR) at the University of Pennsylvania brain bank. Frontal cortex from control and Braak stage VI Alzheimer’s disease cases were used to prepare gray matter, which was homogenized in 3 vol/g of cold High Salt RAB buffer (.75 M NaCl, 100 mM Tris, 1 mM EGTA, 0.5 mM MgSO_4_, 0.02 M NaF, 2 mM DTT, pH 7.4). All buffers were supplemented with protease inhibitor cocktail, phosphatase inhibitor, and HDAC inhibitors (2 uM Trichostatin A and 10 mM nicotinamide). The high-salt RAB homogenates were incubated at 4° C for 20 min to depolymerize MTs, then centrifuged at 100,000 xg for 30 min at 4° C to yield the high-salt (HS) supernatant fraction. Pellets were re-homogenized and centrifuged in 3 vol/g of cold High Salt RAB buffer. Resultant pellets were homogenized in 3 vol/g RIPA buffer (50mM Tris pH 8.0, 150mM NaCl, 1% NP-40, 5mM EDTA, 0.5% sodium deoxycholate, 0.1% SDS) and centrifuged at 100,000g for 30min followed by another RIPA extraction containing 20% sucrose, thus allowing myelin floatation. Finally, the resultant insoluble pellets were extracted in 1 vol/g tissue in urea buffer (7M urea, 2M Thiourea, 4% CHAPS, 30mM Tris, pH 8.5). Tau proteins present in soluble high-salt (HS) and insoluble urea fractions were analyzed by SDS-PAGE electrophoresis and western blotting using tau-5, T46, and K9JA antibodies to detect total tau protein fragmentation patterns.

## Results

### Distinct tau acetylation patterns are observed within the MTBR compared to the adjacent proline-rich region

Cell culture analysis previously identified lysines 163, 280, 281, and 369 as major sites of human tau acetylation (see lysines highlighted in Fig 1A schematic). One of these sites, K163, is localized to the tau proline-rich domain and is present in all 6 tau isoforms expressed in human brain. Recent mass spectrometry analysis identified K163 as a prominent site of acetylation *in vitro*, in cultured cells, and in wild-type mouse brain [3, 5, 8]. Therefore, given the potential significance of K163 acetylation, we sought to generate an acetylation-specific polyclonal tau antibody against a tau peptide antigen containing acetylated residue K163 (termed ac-K163). Double-affinity purification with unmodified and chemically acetylated K163 peptides was employed to enrich for the acetylation-specific antibody pool. We confirmed the site specificity of the ac-K163 antibody by transfecting tau into QBI-293 cells, a cell line that lacks any endogenous tau protein and therefore is amenable to antibody specificity analysis using ectopically expressed tau proteins. Wild-type non-acetylated 2N4R-tau showed no immunoreactivity with ac-K163 under any conditions analyzed, however, tau was readily acetylated at K163 in the presence of ectopically co-expressed CBP, confirming that ac-K163 recognizes acetylated tau (Fig 1B, lane 1). Importantly, 2N4R-tau containing a K163R mutation abolished ac-K163 immunoreactivity (Fig 1B, lane 3), highlighting the site-specificity of ac-K163 for the targeted K163 residue.

We further tested ac-K163 immunoreactivity by western analysis using additional tau isoforms that were expressed in the absence or presence of CBP. All tau proteins analyzed were only ac-K163 immunoreactive in the presence of CBP (Fig 1C). Surprisingly, we observed an isoform-specific acetylation pattern, in which 0N4R tau proteins lacking the N-terminal inserts were more strongly ac-K163 immunoreactive compared to other 3R and 4R tau isoforms (Fig 1C, lane 5). In contrast, acetylation at residue K280 showed no apparent differences in immunoreactivity among the 4R-tau isoforms analyzed, as both 2N4R and 0N4R isoforms were similarly immunoreactive with ac-K280.

*In vitro* assays using recombinant, purified tau proteins in the presence of acetyl-CoA and CBP confirmed CBP-dependent K163 acetylation. Similar to the above cell-based results, purified 0N4R-tau proteins were strongly acetylated at K163 in the presence of CBP, but not upon incubation with acetyl-CoA alone, implying that little detectable auto-acetylation occurs at this distal acetylation site localized to the proline-rich region (Fig 1D, lanes 4–6). *In vitro* acetylation at K163 was less abundant using 3R-tau proteins compared to 4R-tau proteins, suggesting that MTBR repeats influence more distal acetylation at residue K163 (Fig 1D, lanes 7–9). Also similar to the cell-based assay, N-terminal inserts were inhibitory towards *in vitro* acetylation at K163, since among the 4R-tau isoforms tested the strongest ac-K163 immunoreactivity was observed with 0N4R tau (Fig 1D, compare lanes 3 and 6). In contrast, residue K280 showed mild auto-acetylation within the MTBR, as indicated by ~ 25% ac-K280 immunoreactivity in the presence of acetyl-CoA alone, which was strongly induced by the addition of purified CBP, as expected (Fig 1E).

To confirm the preferential auto-acetylation within the MTBR, a tau-K18 fragment comprising only the 4-repeat region itself (~15 kDa fragment) was strongly auto-acetylated in the presence of radiolabeled [^14^C]-acetyl-CoA (Fig 1F, lane 1), however, a full-length tau fragment lacking all 4 repeats (referred to as ΔK18, ~35 kDa fragment) was only acetylated in the presence of CBP (Fig 1F, compare lanes 2–4). Notably, ΔK18 was unable to be acetylated *in trans* in the presence of K18, suggesting predominantly *cis* auto-acetylation, consistent with our previous study [9]. Thus, CBP non-selectively acetylates tau within the proline-rich and MTBR regions (e.g. both K163 and K280), while tau auto-acetylation occurs prominently within the lysine-rich MTBR (e.g. K280). Given the unique auto-acetylation properties of tau, we sought to further explore the consequences of this potentially critical auto-regulatory mechanism.

### Tau auto-acetylation promotes tau proteolytic degradation

**Fig 2:**
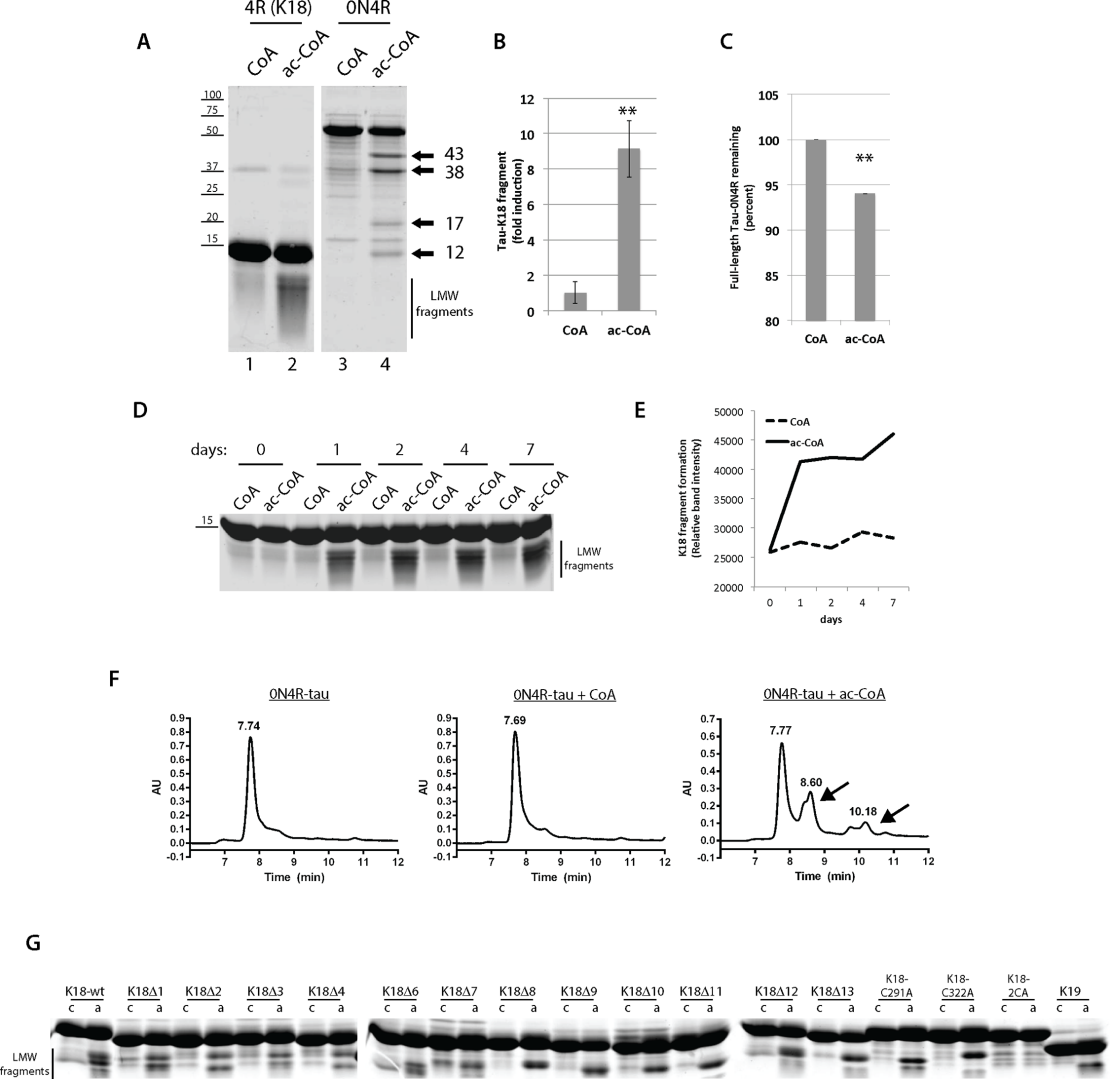
Tau auto-acetylation promotes tau-K18 and full-length tau fragmentation. **A)** Recombinant full length tau (0N4R) or tau-K18 containing only the MTBR repeats were incubated in the presence of CoA or acetyl-CoA for 1 day and reaction products were analyzed by analyzed by SDS-PAGE and Coomassie staining. The fold increase in acetyl-CoA-induced tau-K18 fragments is quantified in **(B)** and the percent remaining full-length 0N4R-tau is quantified in **(C)**, which resulted from N = 3 independent experimental replicates. Double asterisks (**) represent p-value ≤ 0.05 using student t-test. **D)** A time-course analysis of tau-K18 fragmentation was performed in the presence of CoA or acetyl-CoA at the indicated time-points from 0–7 days and tau fragment formation was quantified in **(E)**. **F)** Size-exclusion chromatography (SEC) was used to measure tau fragments generated in the absence of cofactor, or in the presence of CoA or acetyl-CoA. Full-length 0N4R tau protein eluted ~ 7.70 min while lower molecular weight tau fragments were detected as slower eluting protein peaks at ~8.60 and ~10.18 min, which are highlighted by the black arrows. **G)** A sequential tau deletion library containing 10-residue deletions spanning the 126 amino acid tau-K18 fragment (tau-K18Δ1- tau-K18Δ13), or tau cysteine mutants lacking one (C291A or C322A) or both (C291/322A) cysteines were incubated in the presence of CoA or acetyl-CoA for 1 day and analyzed by SDS-PAGE and Coomassie staining. We note that purified tau-K18Δ5 was unstable and therefore was not included in this analysis.

We next examined the effects of *in vitro* tau auto-acetylation on tau fragmentation. Previous studies of bacterial acetyltransferases have hinted that auto-acetylation could be coupled to downstream post-translational processing including proteolysis [25]. Full-length 0N4R or tau containing only the repeat region (tau-K18) were auto-acetylated with acetyl-CoA or incubated with a CoA control for up to 7 days *in vitro*. Surprisingly, we noticed that acetyl-CoA, but not CoA, led to progressive fragmentation of tau-K18 into low molecular weight (LMW) species that were < 10 kDa (Fig 2A, lanes 1–2). Similarly, auto-acetylation of full-length 0N4R tau proteins led to production of distinct tau fragments, however, in contrast to low molecular weight smearing, full-length 0N4R-tau generated distinct ~ 43, 38, 17 and 12 kDa tau fragments (Fig 2A, lanes 3–4, see fragments depicted by arrows). Quantification of these results showed a ~ 10-fold increase in tau-K18 fragmentation (Fig 2B), while 0N4R-tau acetylation led to a mild but significant reduction in full-length protein that coincided with the accumulation of tau fragments (Fig 2C). A time-course analysis indicated that tau-K18 fragments were robustly detected by 1 day incubation *in vitro*, with a small increase in fragmentation thereafter up to 7 days (Fig 2D and 2E).

To confirm that auto-acetylated tau generates bona fide truncation fragments, size-exclusion chromatography (SEC) was employed to detect more slowly eluting low molecular weight tau fragments (Fig 2F). SEC analysis of either untreated or CoA treated 0N4R-tau reactions showed a single prominent full-length tau protein peak eluting at ~ 7.70 min with no apparent smaller molecular weight tau peaks detected. However, incubation of 0N4R-tau under conditions that promote full auto-acetylation in the presence of acetyl-CoA led to the accumulation of distinct N- and C-terminal tau fragments eluting at ~8.60 and ~10.18 min (Fig 2F, see black arrows denoting tau fragments), consistent with the acetylation-induced generation of low molecular weight tau fragments.

Since tau fragmentation has been previously noted *in vitro* [26], we considered the possibility that acetylation could induce intrinsic auto-cleavage events resulting in production of tau fragments. To map the residues required for tau proteolysis, we used a tau-K18 deletion/mutation panel of proteins previously generated [9], which contains sequential 10-residue deletions in tau-K18 (referred to as K18Δ1- K18Δ13) as well as single and double cysteine mutants (C291/C322) that impair tau auto-acetylation. While none of the internal deletion proteins fully abrogated tau fragmentation in a 1 day *in vitro* acetylation reaction, a cysteine-less tau-K18 protein containing C→A substitutions at both C291 and C322 (2CA) was sufficient to abrogate acetylation-induced fragmentation (Fig 2G).

**Fig 3:**
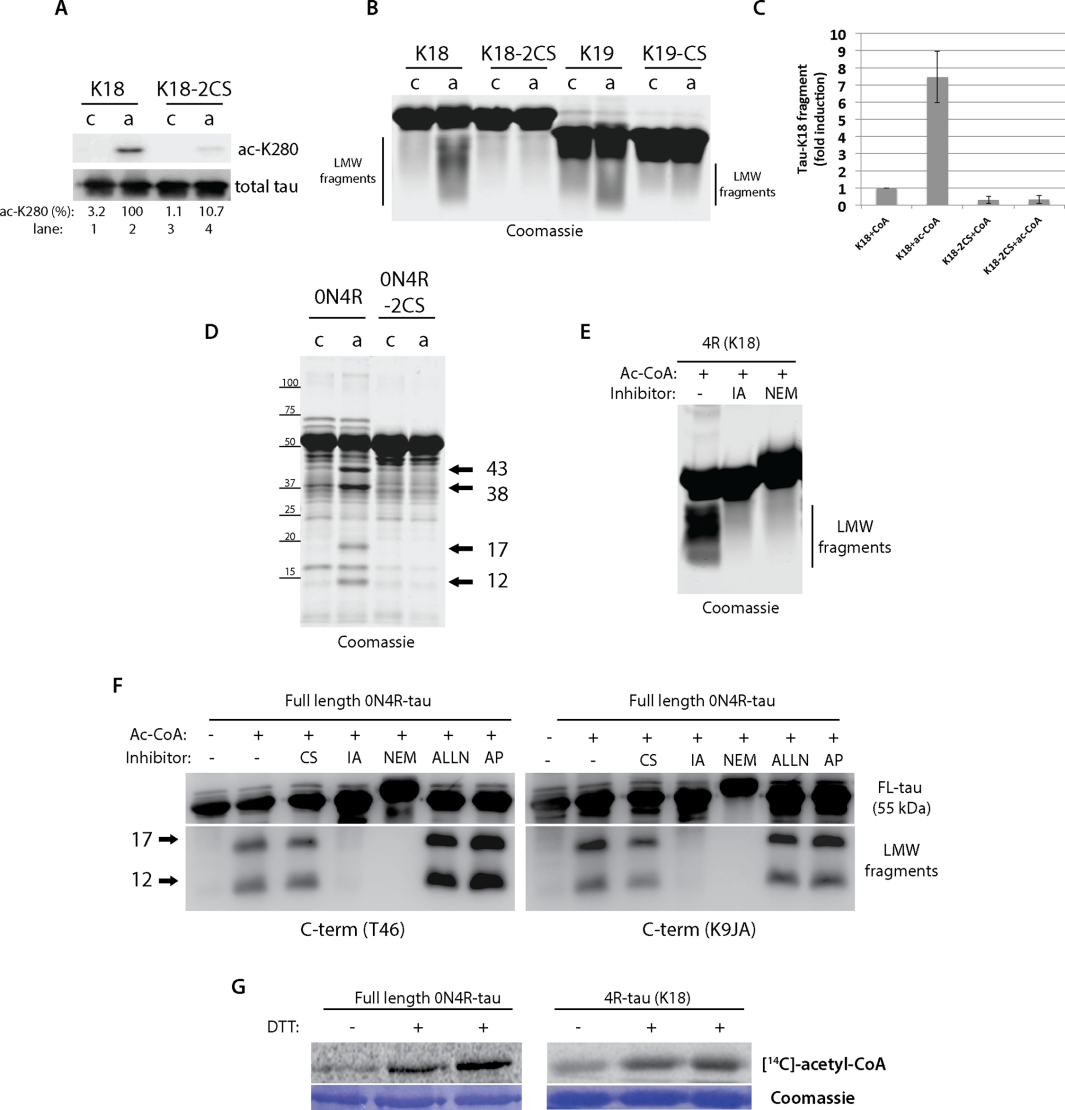
Tau auto-acetylation induced fragmentation requires tau cysteines within the MTBR region. **A)** Tau-K18 or a cysteine-less mutant containing C→S substitutions at C291 and C322 were incubated *in vitro* in acetylation reactions and analyzed by western blotting to examine auto-acetylation using ac-K280 and total tau antibodies (percent ac-K280 immunoreactivity was determined as a ratio of total tau). **B)** Tau-K18, tau-K19, and their corresponding cysteine to serine mutants were incubated with CoA or acetyl-CoA and analyzed by SDS-PAGE and Coomassie staining. Tau-K18 and tau-K19 generated low molecular weight smears (referred to as LMW fragments). **C)** The fold induction of acetyl-CoA-induced fragmentation was quantified from wild-type and cysteine mutant tau-K18 proteins. Note, cysteine mutants completely abolished tau-K18 fragment production. **D)** Acetylation of full-length 0N4R-tau generated a range of N- and C-terminal tau fragments highlighted by black arrows that were reduced with the 0N4R-2CS mutant. **E-F)** Tau-K18 or full-length 0N4R tau were incubated with acetyl-CoA in the absence or presence of the protease inhibitors chymostatin (CS), iodoacetamide (IA), N-ethylmaleimide (NEM), ALLN, or Antipain (AP), and tau-K18 LMW tau fragments **(E)** or the 17/12 kDa fragments generated by 0N4R-tau **(F)** were analyzed by SDS-PAGE and Coomassie staining or immunoblotting using C-terminal T46 and K9JA tau antibodies. **G)** Tau proteins were incubated in acetylation reactions in the absence or presence of 1mM DTT, radiolabeled with [^14^C]-acetyl-CoA, and analyzed by SDS-PAGE and Coomassie staining followed by autoradiography and phosphorimaging.

We therefore generated a panel of cysteine mutant tau proteins and evaluated their ability to undergo acetylation-induced fragmentation. Mutation of both C291 and C322 led to impaired auto-acetylation at residue K280 (Fig 3A), and also dramatically impaired the ability of 4R tau-K18 (2CS) and 3R tau-K19 (single C322S) to generate fragments (Fig 3B). Quantification of these results showed that cysteine mutants completely eliminated the ~10-fold increase in acetylation-induced fragmentation (Fig 3C). Further confirming the cysteine-dependent fragmentation of full-length tau, the formation of ~17 and 12 kDa fragments produced by 0N4R-tau auto-acetylation was abrogated by cysteine mutations (Fig 3D, see 0N4R-2CS). Since mutations of internal tau cysteines prevented tau fragmentation, these results suggest that the observed auto-proteolytic activity is intrinsic to the tau proteins themselves rather than any extraneous non-tau proteases present within the acetylation reactions. We nonetheless considered the possibility that other tau protease activities could be present. However, tau cleavage with increasing concentrations of calpain, a well-characterized and robust tau protease [27], generated an entirely distinct pattern of N- and C-terminal tau fragments (S1 Fig). Specifically, calpain generated prominent ~ 15 kDa N-terminal and ~ 25 kDa C-terminal fragments, suggesting that tau itself possesses a unique auto-proteolytic activity compared to that observed with other tau proteases.

Further supporting the role of tau cysteines, the irreversible cysteine blocking agents iodoacetamide (IA) and N-ethylmaleimide (NEM) were sufficient to prevent fragmentation of tau-K18 (Fig 3E) and 0N4R-tau (Fig 3F) proteins induced by incubation with acetyl-CoA, suggesting free thiol groups mediate acetylation-induced tau fragmentation. Interestingly, only irreversible cysteine protease inhibitors were effective, as the reversible cysteine or serine protease inhibitors chymostatin, ALLN, and antipain did not prevent acetylation-induced tau proteolysis (Fig 3F). We note that the low molecular weight ~17 and 12 kDa fragments are indeed derived from the tau C-terminus, since immunoblotting analysis using T46 and K9JA antibodies (detecting the extreme C-terminus and C-terminal MTBR epitopes, respectively) were strongly immunoreactive with both tau fragments (Fig 3F). Finally, to further support the role for tau cysteines in this auto-catalytic process, we tested whether oxidative reaction conditions lacking the reducing agent dithiothreitol (DTT), which favor cysteine disulfide cross-linking, are sufficient to impair tau auto-actetylation. Indeed, omitting DTT led to slightly reduced tau auto-acetylation of either full-length 2N4R-tau or tau-K18 proteins, as assessed by pan-[^14^C]-acetyl-CoA labeling (Fig 3G).

### Tau auto-proteolytic cleavage induced by acetyl-CoA maps to within the 2^nd^ and 4^th^ MTBR

**Fig 4:**
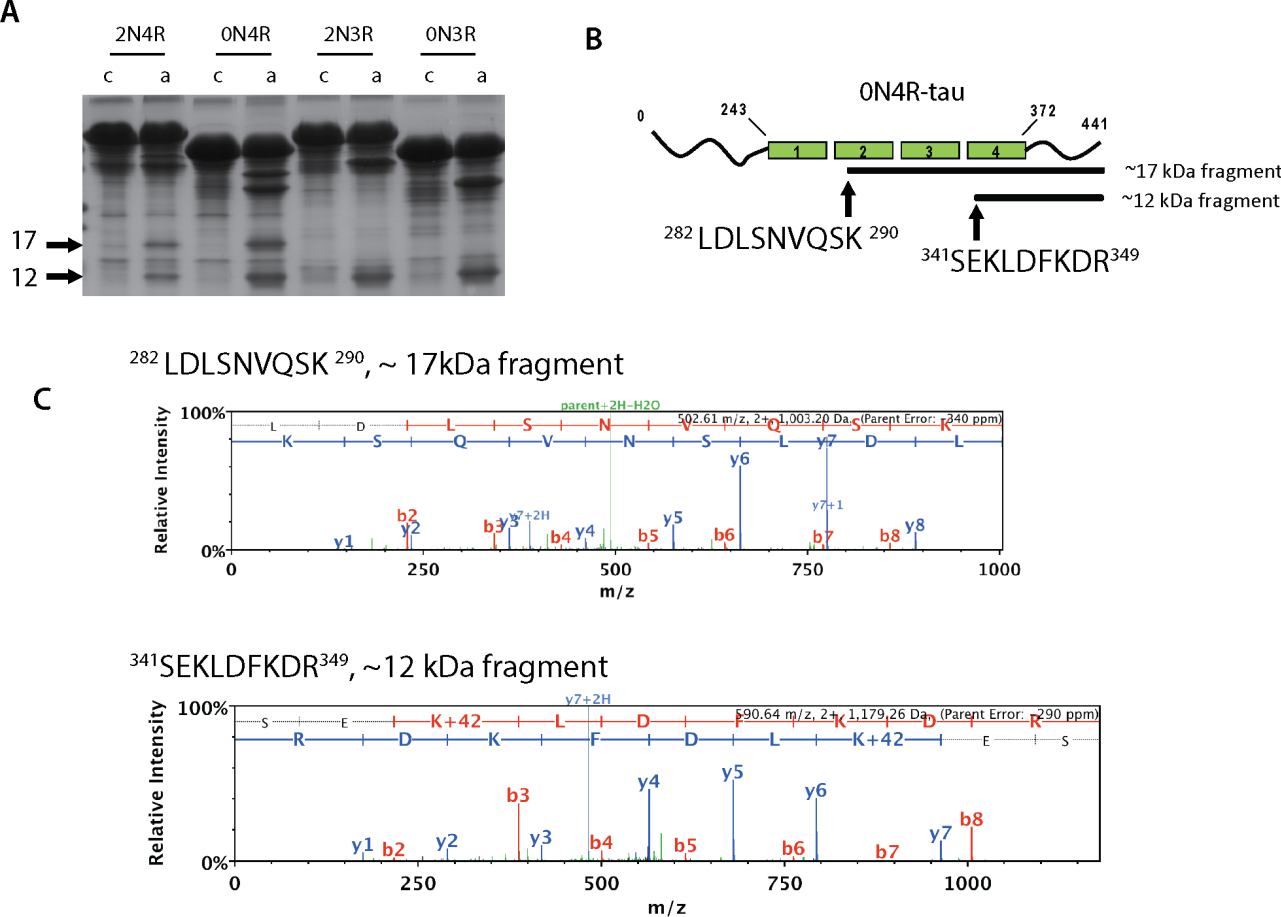
Acetylation-induced tau ~17 and ~12 kDa fragments map to the 2^nd^ and 4^th^ MTBR domains, respectively. **A)** Large-scale 2N4R, 0N4R, 2N3R, or 0N3R tau proteins were incubated with CoA or acetyl-CoA and analyzed by Coomassie staining to detect tau fragments by Coomassie staining. Using large scale reactions as input, C-terminal fragments were immunprecipitated using anti-tau T46 antibodies and subject to mass spectrometry analysis. **B)** Extreme N-terminal peptides were mapped by mass spectrometry and were localized to the start of the 2^nd^ (17 kDa fragment) and 4^th^ (12kDa fragment) MTBR, and the peptide spectra matching these extreme N-terminal sequences are depicted in **(C).**

In order to map the acetylation-induced cleavage sites, larger scale reactions containing full-length tau isoforms were auto-acetylated (Fig 4A). Full-length 4R-tau isoforms containing both cysteines (C291 and C322) generated both ~17 and 12 kDa fragments, while 3R-tau isoforms containing only C322 generated only the 12 kDa fragment, implying that tau cysteine residues direct the cleavage specificity towards one or both C-terminal fragments. Both fragments were immunoprecipitated from reactions containing acetylated 0N4R tau using the C-terminal T46 antibody, and protein bands corresponding to the tau fragments were excised for analysis by mass spectrometry. Analysis of extreme N-terminal peptides derived from each fragment identified ^282^LDLSNVQSK^290^ (2^nd^ MTBR) at the start of the 17 kDa fragment and ^341^SEKLDFKDR^349^ (4^th^ MTBR) at the start of the 12 kDa fragment (Fig 4B and 4C). Additional efforts to N-terminally sequence the tau fragments were not successful. However, we note that the experimentally determined cleavage sites based on our mass spectrometry analysis predicts molecular masses of 16.9 and 10.7 kDa C-terminal fragments, which is in close agreement with the observed ~17 and 12 kDa fragments by western and SDS-PAGE gel analysis. Thus, our results indicate that acetylation-induced cleavage requires tau catalytic cysteine residues, thereby generating larger tau N-terminal fragments and their corresponding ~17 and ~12 kDa C-terminal fragments containing portions of the MTBR domains.

**Fig 5:**
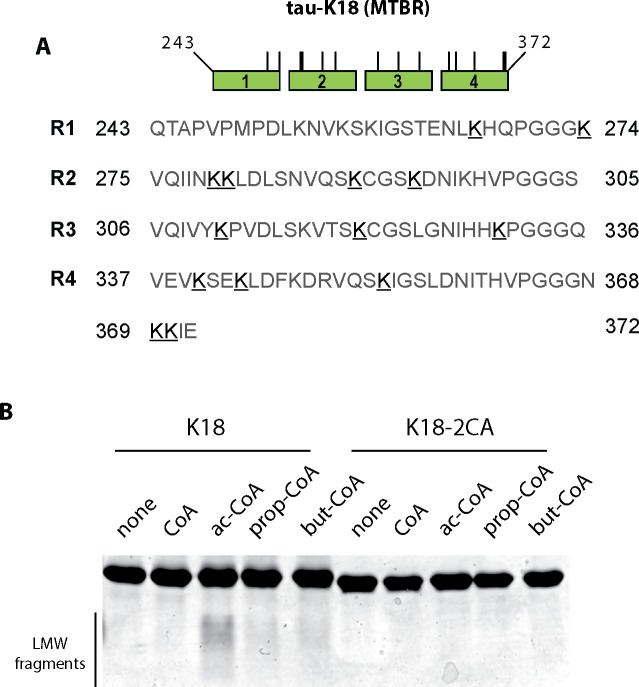
Tau auto-acetylation induced fragmentation shows specificity for acetyl-CoA compared to longer acyl-CoA derivatives. **A)** Tau-K18 or the cysteine-less tau-K18-2CA mutant were incubated with a panel of acyl-CoA derivatives of varying lengths (CoA, acetyl-CoA, butyryl-CoA, and propionyl-CoA) and acylated tau proteins were analyzed by mass spectrometry to map butyrylated and propionylated tau lysine residues. The tau-K18 schematic illustrates the positions corresponding to lysine butyrylation (+70) and lysine propionylation (+56) sites that were detected within the 4-repeat MTBR. Positively identified lysines (K’s) are bolded and underlined (see also S1 Table). **B)** Modified tau proteins were analyzed by Coomassie staining to assess tau fragmentation. We note that tau fragmentation is cysteine-dependent and occurs most readily with acetyl-CoA substrate compared to longer acyl-CoA derivatives.

To assess the specificity of acetyl-CoA in mediating tau auto-proteolytic cleavage, we performed reactions with the related acyl-CoA derivatives butyryl-CoA and propionyl-CoA, which are similar but slightly longer than acetyl-CoA and also capable of modifying target lysines via butyrylation and propionylation reactions, potentially linking cellular metabolic processes to acylation of target lysines [28–30]. To determine whether tau proteins are indeed subject to modification by these other short chain CoAs, *in vitro* reactions containing propionyl-CoA and butyryl-CoA derivatives were analyzed by mass spectrometry and modified lysines detected by +56 (propionylation) or +70 (butyrylation) mass additions were identified (S1 Table) within the 4-repeat containing tau-K18 sequence (Fig 5A, modified lysine residues are underlined). Many of the identified lysine residues are also acetylation targets [3, 5], suggesting common overlap among acetylated, butyrylated, and propionylated acceptor lysines. Analysis of all acylated tau-K18 proteins on Coomassie gels revealed that acetyl-CoA was able to promote accumulation of low molecular weight tau fragments, which were not readily observed with other acyl-CoA derivatives (Fig 5B). Thus, we conclude that, while larger acyl-CoA derivatives are sufficient to modify tau via propionylation and butyrylation, they are not as efficient as acetylation in generating auto-proteolytic processed tau fragments *in vitro*.

**Fig 6:**
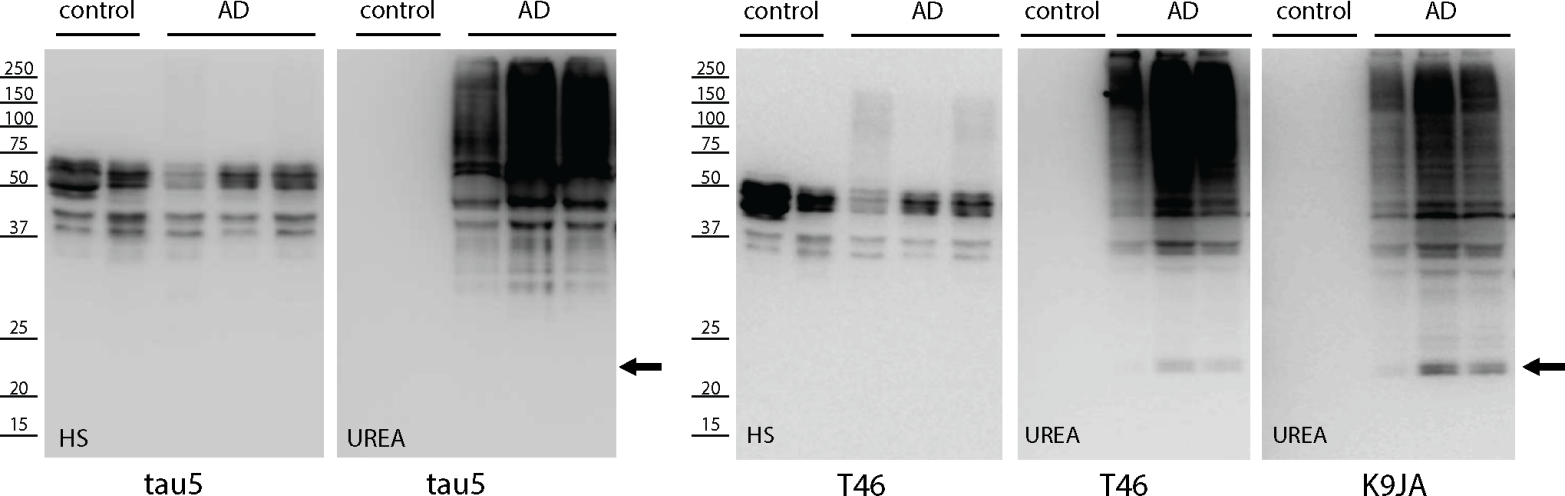
The generation of ~24 kDa but not ~17 and 12 kDa C-terminal tau fragments in AD brain. Gray matter from whole frontal cortex (~5g) from control and AD brain was sequentially extracted with buffers of increasing strength (see brain extraction methods) to yield soluble and insoluble tau fractions containing normal and aggregated tau species. Brain homogenates were analyzed by immunoblotting using N-terminal (tau5) or C-terminal (T46 and K9JA) antibodies. We note that none of the cases examined contained ~17/12 kDa LMW tau fragments, however, we detected ~24 kDa C-terminal tau fragments indicated by the black arrow, which is consistent with a previous study [31].

To examine tau proteolysis in cells, full-length 2N4R-tau was co-expressed with CBP to promote full tau acetylation, and we observed enhanced accumulation of C-terminal proteolytic tau smearing with a panel of tau antibodies, which was dependent on CBP acetyltransferase activity since this effect was reduced with an enzymatically inactive CBP-LD mutant (S2 Fig). To examine if C-terminal tau fragments accumulate in AD brain, we sequentially extracted frontal cortex brain tissue from control and AD cases (Fig 6). Western analysis of high salt (HS) extracted brain homogenates containing soluble tau isoforms showed the expected migration pattern of human tau isoforms ~ 50–60 kDa, as detected with both N-terminal (tau5) and C-terminal (T46) tau antibodies. Insoluble brain homogenates were generated from high-salt insoluble material, which contained characteristic high molecular weight tau species corresponding to insoluble tau aggregates. However, we also noted a ~ 24 kDa low molecular weight tau fragment that was specifically detected in AD brains using the C-terminal T46 and K9JA antibodies, but not the N-terminal tau5 antibody (Fig 6, see black arrowhead). Although we found little evidence for ~17 or ~12 kDa C-terminal tau fragments in the late-stage AD brains analyzed, the presence of a slightly larger ~ 24 kDa fragment is consistent with a recent study [31], and supports the presence of cleaved C-terminal fragments containing a portion of the tau MTBR that are detectable and stabilized in AD brain.

## Discussion

In this study, we provide the first evidence that tau possesses an intrinsic auto-catalytic activity that facilitates acetyl group transfer and self-fragmentation. Using biochemical, cellular, and mass spectrometry approaches, we provide evidence for a catalytic mechanism in which 4R-tau containing C291 and C322 mediates full acetylation-induced proteolysis, as either mutation or pharmacological blockade of cysteines prevents the auto-proteolytic cascade. Our study suggests a potential link between acetylation and downstream tau processing, which may contribute to the heterogeneous accumulation of N- and C-terminal tau fragments that have been widely detected in mouse and human AD brain as well as CSF from a range of tauopathy patients [31–37].

While acetylation is an energetically favorable process that could be vulnerable to false positive identification of acetylated residues [19, 38], our results suggest that tau auto-acetylation is a regulated process. The requirement for cysteines (Fig 3A) suggests a catalytic mechanism involving an acetyl-cysteine intermediate and subsequent acetyl group transfer to lysine residues, similar to other cysteine-dependent acetyltransferases including members of the MYST [18], NAT [39, 40], and YopJ families [41–43]. This contrasts with direct acetylation of targeted lysine residues mediated by CBP/p300. Secondly, we observed that auto-acetylation does not uniformly occur on lysines spanning the tau protein, but rather showed site-specificity within the lysine-rich MTBR domain (Fig 1). Previous studies suggest that auto-acetylation of cellular substrates can occur prominently at basic stretches of lysine residues, of which the tau MTBR could be an ideal candidate [21]. Thus, when free in solution, the tau MTBR could undergo cysteine-dependent auto-acetylation in the presence of physiological concentrations of acetyl-CoA. This activity may be suppressed when tau is bound to tubulin or MTs, but activated upon tau dissociation during pathological conditions that abrogate tau-MT interactions, thus allowing acetyl-CoA accessibility to free tau cysteines [9].

Although the exact mechanism by which tau auto-acetylation leads to fragmentation remains unclear, an apparent non-enzymatic tau cleavage was previously observed by Watanabe *et al*. using tau proteins incubated up to 90 days *in vitro* [26]. The authors identified asparagine residues within the MTBR as the likely sites of auto-proteolytic cleavage, leading to disappearance of full-length tau and concurrent high molecular weight tau smearing. Although it is possible that acetylation accelerates a similar process to achieve rapid tau cleavage by ~ 1–2 days in our study, we find no evidence for high molecular weight smearing, at least at the time-points and under the conditions employed in our study. Furthermore, the ~17 and ~12 kDa C-terminal fragments generated from full-length tau are not suggestive of cleavage at asparagine residues, or by fragmentation mediated by other tau proteases such as cathepsins or thrombin, although we cannot rule out this possibility [44, 45]. Lastly, we find no evidence for contaminating proteases that may be present in our tau protein preparations (S1 Fig), which are fully heat denatured and stringently purified using chromatography methods.

It is plausible that tau possesses intrinsic dual acetyltransferase/protease activities, in a manner similar to bacterial effector proteins including YopJ family members that utilize cysteine-dependent catalysis to facilitate either acetylation, proteolysis, or in some cases both activities [41–43, 46]. Indeed, seemingly distinct cysteine protease and acetyltransferase activities within the YopJ family are achieved by a common catalytic mechanism in which a catalytic triad of residues facilitates either peptide hydrolysis or acetyl group transfer by employing a well conserved charge relay system. In fact, some YopJ/HopZ members are reported to perform numerous independent biochemical activities as either proteases or acetyltransferases [25]. Whether tau possesses these catalytic functions *in vivo* in either an auto-regulatory manner or perhaps directed towards non-tau substrates is not known. Our results indicate that different tau isoforms may possess different affinities to undergo auto-acetylation and proteolysis, with full-length 4R-tau isoforms showing the most robust acetylation and production of both 17 and 12 kDa C-terminal fragments, while 3R-tau produced only the 12 kDa fragment (Fig 4A) [9]. In addition, the exact function for tau N-terminal inserts has not been fully elucidated. Our data suggest a potential inhibitory role for these domains in tau acetylation, as 0N4R tau isoforms showed the most robust ac-K163 immunoreactivity, implying that N-terminal repeats may significantly influence tau acetylation status.

Since tau auto-acetylation occurs via a cysteine-dependent mechanism, conceivably, more specific and less toxic compounds than NEM and IA (Fig 3D and 3E) that engage tau free cysteines may represent therapies to prevent auto-acetylation and downstream cleavage. In this regard, methylene blue [47–54], and more recently 1,2-dihydrobenzene [55], were shown to interact with tau cysteines and also alleviated cognitive deficits in tau transgenic mouse models. Whether these compounds act via inhibition of tau auto-acetylation remains unknown. If so, inhibition of tau acetylation could provide a simple rationale for why cysteine interacting compounds may confer neuroprotection against tau-mediated toxicity.

Based on our results, we hypothesized that acetylation-induced tau C-terminal fragments containing portions of the MTBR might accumulate in AD brain and potentially seed tau aggregation or alter synaptic signaling, for example by modulating the recently described KIBRA protein leading to memory loss [7]. Our analysis in AD brain detected the presence of a ~ 24 kDa C-terminal tau fragments, similar to that observed recently [31], implying that smaller tau fragments < 24 kDa are either below our detection sensitivity or that only larger MTBR fragments accumulate in AD brain. We cannot exclude the possibility that ~17 and/or ~12 kDa fragments are indeed generated *in vivo*, but perhaps further processed by additional modifications (e.g. phosphorylation, ubiquitination) to yield slightly larger molecular mass fragments. Alternatively, smaller C-terminal tau fragments could be targeted for degradation *in vivo*. In this regard, we note that mass spectrometry analysis of the ~12 kDa tau fragment identified an extreme N-terminal sequence ^341^SEKLDFKDR^349^ that is immediately adjacent to a putative recognition motif for chaperone-mediated autophagy (CMA), ^336^QVEVK^340^ [56]. Thus, it is conceivable that tau auto-proteolysis exposes a putative CMA targeting sequence as a clearance mechanism to degrade potentially toxic tau fragments, a process that may become impaired in tauopathies. Future experiments in cell-based models could clarify whether MTBR fragments accumulate intracellularly or perhaps become secreted, and whether autophagy is activated in response to tau auto-proteolytic activity. In addition, efforts to analyze AD brains at different Braak stages could further clarify whether tau fragmentation occurs earlier in AD pathogenesis. If so, fragments may be generated continually but targeted for degradation due to their potential for toxicity.

In summary, we provide evidence for a novel acetylation-induced auto-proteolytic cascade that generates N- and C-terminal tau fragments with potential implications for normal tau physiology and its pathological aggregation. Future efforts to characterize the disease relevance of tau auto-regulation could provide unique opportunities to directly modulate tau catalytic functions as a new therapeutic avenue for AD and other tauopathies. Tau auto-proteolytic fragmentation could also provide opportunities for biomarker discovery in tauopathies characterized by the accumulation of tau fragments in CSF and other patient biofluids.

## Supporting Information

S1 Fig Calpain-mediated tau proteolysis *in vitro*. Full-length 2N4R tau proteins were incubated with increasing concentrations of recombinant calpain-2 (0-.5U enzyme/reaction). Reactions containing calpain-induced tau fragments were analyzed by immunoblotting using a panel of tau antibodies detecting N-terminal (T14 and tau-5 antibodies) or C-terminal (K9JA and T46) tau epitopes. The calpain-mediated fragmentation patterns defined by the 15 kDa N-terminal fragment and 25 kDa C-terminal fragment are distinct from tau auto-acetylation induced fragmentation (Fig 2).
(TIF)

S2 Fig Acetylation-induced tau proteolysis in cultured cells. CBP-catalyzed tau acetylation was performed in co-transfected QBI-293 cells. Cell lysates were analyzed by tau immunoblotting using T46, K9JA, and tau-5 antibodies. Proteolytic tau low molecular weight smears were observed upon full tau acetylation in the presence of wild-type CBP, but were reduced with an enzymatically inactive CBP mutant (CBP-LD).
(TIF)

S1 Table Mass spectrometry-based identification of sites of tau propionylation and butyrylation within the MTBR. Tau *in vitro* acylation reactions were performed for 1hr at 37° using propionyl-CoA and butyryl-CoA, slightly longer acyl-coA derivatives compared to acetyl-CoA, and 1.0 μg reaction products were resolved by gel elecrophoresis and analyzed by mass spectrometry (see methods). Unambiguously modified lysines were identified and sorted by location within the tau-K18 protein sequence. The identified lysines shown in column 2 correspond to lysines that are underlined in Fig 5A.
(XLS)
