# Supplementary figures and images for: Intrinsic Tau Acetylation Is Coupled to Auto-Proteolytic Tau Fragmentation
Source: PLoS One. 2016 Jul 6;11(7):e0158470. doi: 10.1371/journal.pone.0158470 (PMC4934699; doi:10.1371/journal.pone.0158470)

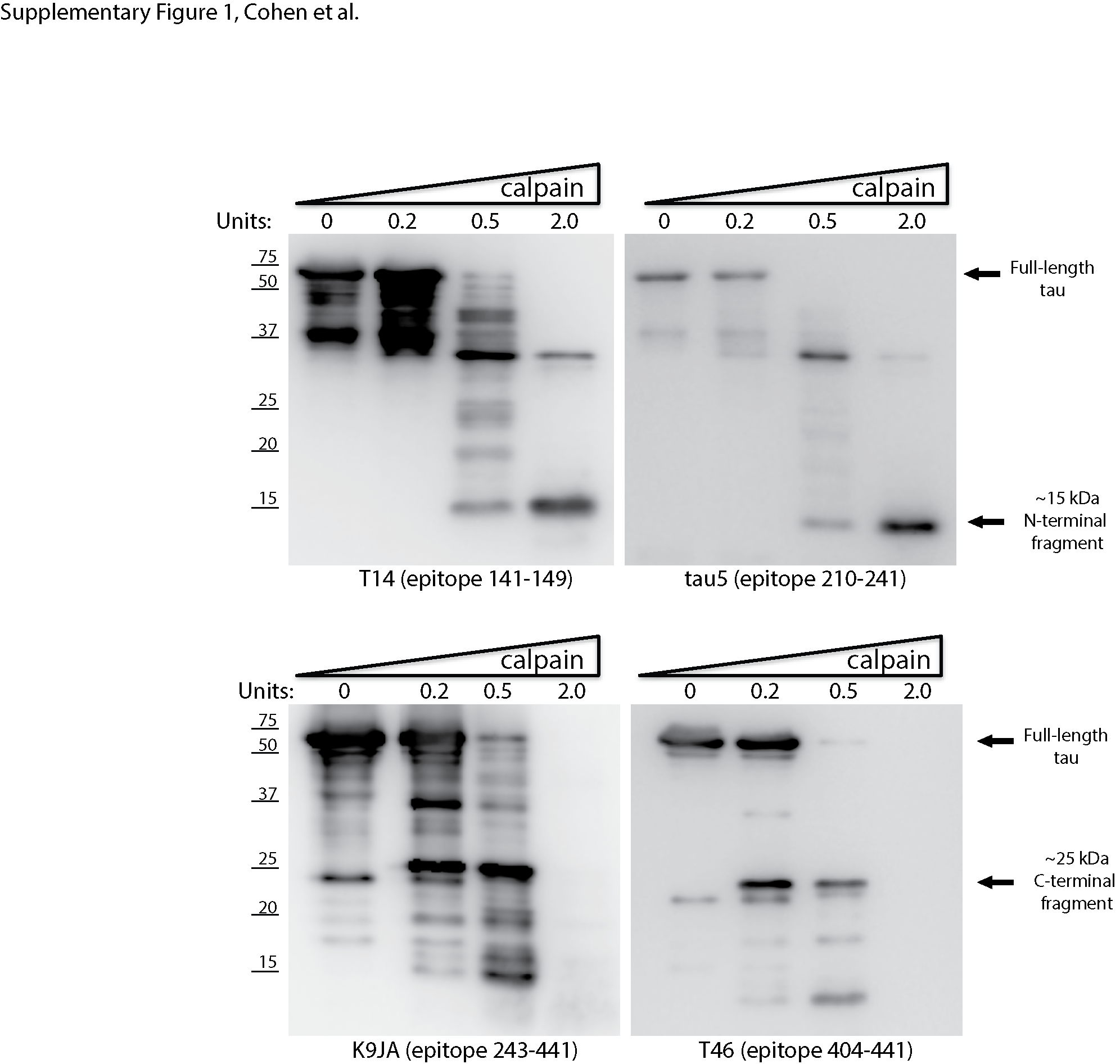

Supplement: S1 Fig — Full-length 2N4R tau proteins were incubated with increasing concentrations of recombinant calpain-2 (0-.5U enzyme/reaction). Reactions containing calpain-induced tau fragments were analyzed by immunoblotting using a panel of tau antibodies detecting N-terminal (T14 and tau-5 antibodies) or C-terminal (K9JA and T46) tau epitopes. The calpain-mediated fragmentation patterns defined by the 15 kDa N-terminal fragment and 25 kDa C-terminal fragment are distinct from tau auto-acetylation induced fragmentation (Fig 2). (TIF) [file pone.0158470.s001.tif]

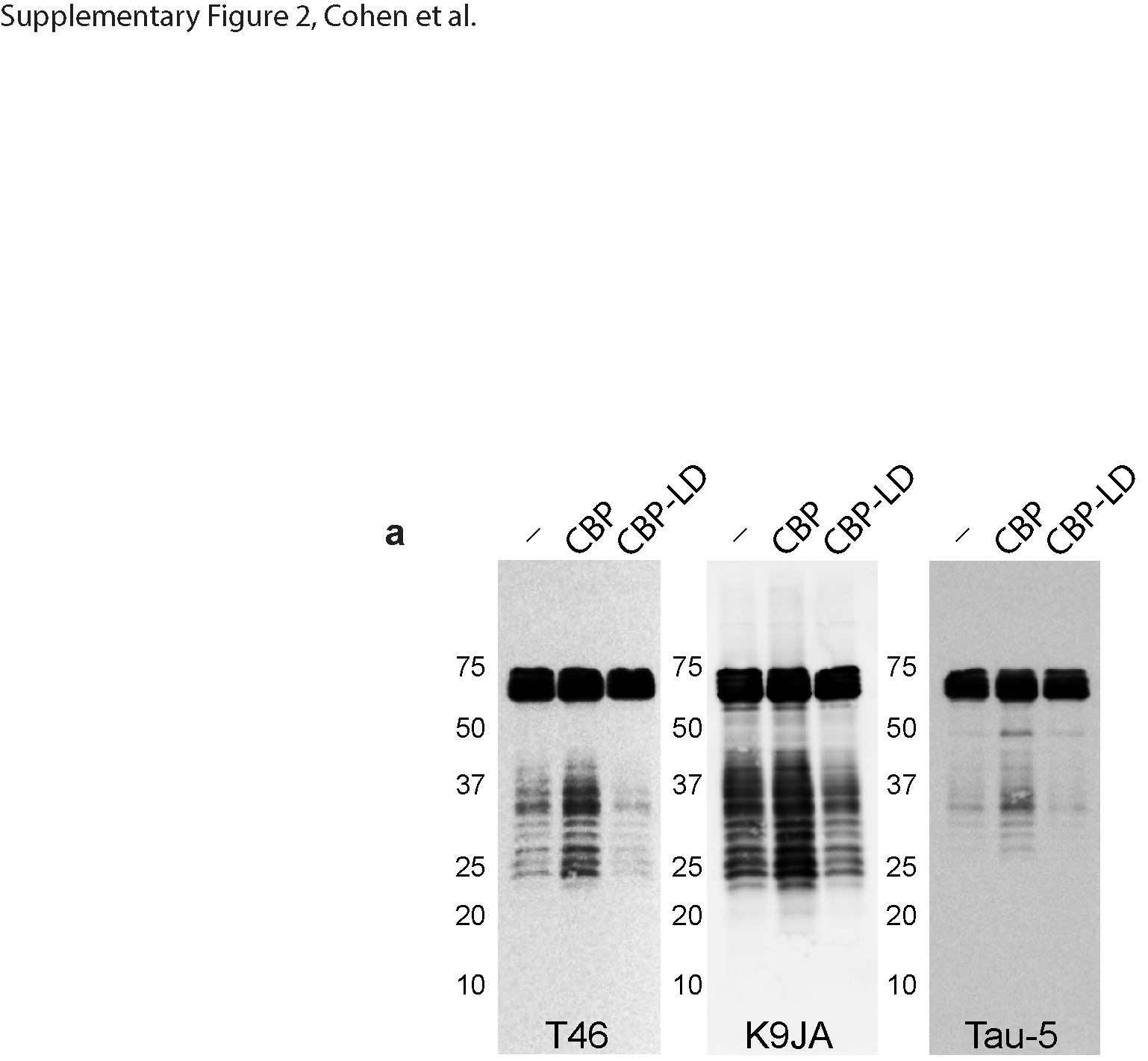

Supplement: S2 Fig — CBP-catalyzed tau acetylation was performed in co-transfected QBI-293 cells. Cell lysates were analyzed by tau immunoblotting using T46, K9JA, and tau-5 antibodies. Proteolytic tau low molecular weight smears were observed upon full tau acetylation in the presence of wild-type CBP, but were reduced with an enzymatically inactive CBP mutant (CBP-LD). (TIF) [file pone.0158470.s002.tif]
